# Circ-phkb promotes cell apoptosis and inflammation in LPS-induced alveolar macrophages via the TLR4/MyD88/NF-kB/CCL2 axis

**DOI:** 10.1186/s12931-024-02677-6

**Published:** 2024-01-29

**Authors:** Xuxia Wei, Xiaomeng Yi, Jianrong Liu, Xin Sui, Lijuan Li, Mei Li, Haijin Lv, Huimin Yi

**Affiliations:** 1 Surgical Intensive Care Unit, The Third Affiliated Hospital of Sun Yat-Sen University, No. 600, Tianhe Road, Tianhe District, Guangzhou, Guangdong 510630 China; 2 VIP Healthcare Center, The Third Affiliated Hospital of Sun Yat-Sen University, No. 600, Tianhe Road, Tianhe District, Guangzhou, Guangdong 510630 China

**Keywords:** Circ-phkb, TLR4, MyD88, NF-kB, Acute lung injury

## Abstract

**Background:**

Circular RNAs (CircRNAs) have been associated with acute lung injury (ALI), but their molecular mechanisms remain unclear.

**Methods:**

This study developed a rat model of lipopolysaccharide (LPS)-induced ALI and evaluated the modeling effect by hematoxylin and eosin staining, Masson’s trichrome staining, lung wet-to-dry weight ratio, terminal deoxynucleotidyl transferase UTP nick end labeling (TUNEL), and enzyme-linked immunosorbent assay (ELISA) detection of inflammatory factors (interleukin-1β, tumor necrosis factor alpha, and interleukin-6). Using lung tissues from a rat model of LPS-induced ALI, we then conducted circRNA sequencing, mRNA sequencing, and bioinformatics analysis to obtain differential circRNA and mRNA expression profiles as well as potential ceRNA networks. Furthermore, we performed quantitative real-time polymerase chain reaction (qRT-PCR) assays to screen for circ-Phkb in ALI rat lung tissues, alveolar macrophages, and LPS-induced NR8383 cells. We conducted induction with or without LPS with circ-Phkb siRNA and overexpression lentivirus in NR8383. Cell Counting Kit-8, C5-Ethynyl-2′-deoxyuridine (Edu), TUNEL, and cytometry were used to identify proliferation and apoptosis, respectively. We detected inflammatory factors using ELISA. Finally, we used Western blot to detect the apoptosis-related proteins and TLR4/MyD88/NF-kB/CCL2 pathway activation.

**Results:**

Our results revealed that both circRNA and mRNA profiles are different from those of the Sham group. We observed a significant circ-Phkb upregulation in NR8383 cells and LPS-exposed rats. Apoptosis and inflammation were greatly reduced when circ-Phkb expression was reduced in NR8383 cells, cell proliferation was increased, and circ-Phkb overexpression was decreased.

**Conclusions:**

In terms of mechanism, circ-Phkb suppression inhibits CCL2 expression via the TLR4/MyD88/NF-kB pathway in LPS-induced alveolar macrophages.

**Supplementary Information:**

The online version contains supplementary material available at 10.1186/s12931-024-02677-6.

## Introduction

Acute lung injury (ALI) is characterized by acute inflammation and tissue damage that causes neutrophil and macrophage dysfunction, alveolar-capillary membrane barrier destruction, and inflammatory pulmonary edema, which ultimately results in respiratory failure [1]. The lung is a continuous immune organ that is frequently exposed to antigens. Pathogen /damage-related molecular patterns can activate related pathways when microbial or aseptic trauma occurs, causing a storm of inflammatory factors and damaging tissues and organs. However, the mechanism that causes ALI remains unclear.

Macrophages are a type of cells mediated by innate immunity and adaptive immunity. They remain in a static state under physiological conditions. They can be recruited to the inflammatory site, when inflammation occurs, to activate immunity through pro-inflammatory factor synthesis and secretion. However, excessive macrophages and increased immune activation have harmful effects, which are not conducive to the timely suspension of anti-inflammatory effects and can result in overwhelming immunity causing inflammatory cytokine storm, leading to acute and chronic lung tissue injury and fibrosis [2–4]. Alveolar macrophages, located on the surface of the alveolar space lumen, are the main white blood cells in the lungs and function by initiating and resolving the immune response in the lungs [5, 6]. Evidence revealed that a decrease in alveolar macrophages improves acute ventilator-induced lung injury in rats and that the immune microenvironment shaped by alveolar macrophages promotes ALI [7, 8]. Therefore, exploring the role of alveolar macrophages in ALI is greatly significant.

Circular RNA (circRNA) is a single-strand circRNA formed in the process of transcription and back splicing. It has an upstream donor site and a downstream adopter site. Circular RNA has been verified to be involved in various physiological processes, including autophagy regulation, apoptosis, and cell proliferation. Dysregulation of its expression may cause disease occurrence [9]. Notably, lung macrophage circRNA is also involved in ALI regulation. A series of circRNAs were differentially expressed in lung macrophages in ALI mice [10–12]. In particular, a recent study revealed evidence that circ_0054633 regulates ALI inflammation, while the protective effect of drug treatment on ALI is closely related to the recovery of mmu_circ_0001679 and circ_0001212 expressions, indicating the important role of circRNA in the disease process of ALI [13, 14]. However, few studies still focus on the function of circRNA in macrophages in the context of ALI.

Therefore, this study developed the LPS-induced ALI rat model and the LPS-induced NR8383 cell inflammation model to explore the function of circRNA in ALI and expand the understanding of ALI from the perspective of circRNA.

## Methods

### Cell culture and treatment

We purchased the rat alveolar macrophage cell line NR8383 from Cellcook (Guangzhou, China) and cultured it in Dulbecco’s Modified Eagle Medium supplemented with 10% fetal bovine serum. Cells were grown at 37℃ in a humidified incubator supplied with 5% CO_2_. To establish an in vitro ALI model, LPS (100 ng/mL) was applied to NR8383 cells, followed by incubation for 48 h.

We used bronchoalveolar wash to obtain alveolar macrophages. After exsanguinations, the lung and trachea were jointly removed and five times rinsed in a 10 mL (4℃) cold saline solution. The cell suspension was centrifuged at 300 × g for 7 min, and the cell pellet was resuspended in RPMI1640 culture media containing 10% fetal calf serum, glutamine (2 mM), penicillin (100 U/mL), and streptomycin (100 µg/mL). We cultured 3 × 10^6^ cells in 6 well plates at 37℃ in an air/CO_2_ (95:5) gas phase. We removed the non-adherent cells after a 4 h attachment time by shaking. More than 95% of the adhering population were alveolar macrophages.

### Animals and ALI model construction

We purchased 12 SD rats (~ 300 g) from Guangdong Medical Laboratory Animal Center and randomly assigned them to two groups (Sham and LPS group), one receiving Sham (phosphate-buffered saline [PBS]) treatment and the other LPS (2 mg/kg, Sigma, MO, USA) induction through an intratracheal spray. Rats were then returned to the cages and were free of food and clean water. Rats were anesthetized and killed after 24 h. Lung tissues were resected for subsequent experiments. The Forevergen Biosciences Animal Center approved all the animal experiments.

### Lung wet-to-dry (W/D) weight ratio

The left lungs (*n* = 3) were immediately weighed after the rats were sacrificed and were then subjected to desiccation at 80℃ for 72 h to determine the dry weight. We calculated the lung W/D ratio by dividing the wet weight by the dry weight.

### Pathological observation and score evaluation

The right lungs (*n* = 3) were taken after the rats were euthanized, treated in 4% formaldehyde, then embedded in paraffin and sliced at 5 μm thickness, and stained with hematoxylin and eosin (H&E) staining (Hematoxylin-Eosin Staining Kit, Solarbio) and Masson’s trichrome staining (Masson’s Trichrome Stain Kit, Polysciences Inc). Lung morphologic changes were observed by light microscopy. A highly experienced and competent pathologist performed all analyses. Lung injury and lung fibrosis were scored as previously described [15, 16].

### Determination of inflammation factors

We collected bronchoalveolar lavage fluid (BALF) from the lungs to investigate the levels of inflammatory factors. This was performed by infusing the lung three times with 2 mL of sterile saline. BALF samples were centrifuged at 12,000 × g at 4℃ for 10 min. We collected and analyzed the supernatants to detect the expression of interleukin (IL)-1β, tumor necrosis factor alpha (TNF-α), and IL-6 by enzyme-linked immunosorbent assay (ELISA) kits (Beyotime, Shanghai, China) following the manufacturers’ instructions.

### RNA isolation and RNA-seq

Lung tissues of Sham and LPS-induced rats were ground in liquid nitrogen. TRIzol reagent (Invitrogen, MA, USA) was used to extract total RNA from lung tissues. After quality validation, total RNA was subjected to RNA-seq. The cDNA library was first prepared using the SmartPCR cDNA kit (CLONTECH Laboratories, Japan). Adaptors were then removed using RsaI digestion (Thermo Fisher Scientific), fragmented, and profiled with Agilent Bioanalyzer. After quality verification using an Agilent Bioanalyzer 2100, cDNA libraries were sequenced on an Illumina HiSeq2000 perform.

### Bioinformatic analysis

We identified the differentially expressed circRNAs and mRNAs using the R software (3.1 version) based on relative levels from next-generation RNA sequencing. The relative RNA expression was determined using the Cufflinks 2.0.2 software. Significantly differently expressed circRNA and mRNA were defined as a fold change of ≥ 2.0 and a false discovery rate of < 0.001.

We connected the differentially expressed circRNAs and mRNAs with miRNAs that were predicted to have binding sites to circRNAs or mRNAs using Miranda software (3.3 version) and Cytoscape (3.2.1 version) to generate a circRNAs-miRNAs-mRNAs interaction network. We performed Gene Ontology (GO) and Kyoto Encyclopedia of Genes and Genomes (KEGG) platform (http://www.genome.jp/kegg/) using KOBAS3.0 (http://kobas.cbi.pku.edu.cn/kobas3/?t=1) to predict the potential role of deferentially expressed circRNAs and mRNAs.

### CircRNA identification and expression quantification

Total RNA was isolated using TRIzol reagent (Invitrogen, Thermo Fisher Scientific) and transcripted to cDNA using the One Step TB Green® PrimeScript™ PLUS RT-PCR kit (Takara, Dalian, China). Genomic DNA (gDNA) was isolated using EasyPure® Genomic DNA Kit (TransGEN, Beijing, China) for circRNA identification. We amplified gDNA and cDNA using divergent and convergent primers and the resultant production was purified and subjected to Sanger sequencing to identify the back splicing site. We amplified cDNA using TB Green® Premix Ex Taq™ II (Takara, TOKO, Japan) with specific circRNA primers to detect the relative expression of circRNA. Table 1 lists the primers used for polymerase chain reaction (PCR) and quantitative real-time PCR (qRT-PCR).

**Table 1:** The information of primers used in this study

Primers name	Primers sequence (5’to3’)	Product size(bp)
rat-circ-Herc1-CF	AACCTTTCCCTGTAGTGGGC	181
rat-circ-Herc1-CR	GCCTCACCGGTCCATTAAGA	
rat-circ-Herc1-LF	GAAACGTGGACAATGCGGAG	165
rat-circ-Herc1-LR	AACGTGGCCACTGCTTATGT	
rat-circ-Smtnl2-CF	CCATGGCTGAAGCATGGAACA	135
rat-circ-Smtnl2-CR	GATGAGGCTTGGACCTCAGA	
rat-circ-Smtnl2-LF	CAGATCCTGCTCGAGTGGTG	165
rat-circ-Smtnl2-LR	CTGCCTTTGTGTGGGACTCA	
rat-circ-Tlk2-CF	CCACAACGTCGAGTAGAACAGC	145
rat-circ-Tlk2-CR	CCCTTGGCGCTAACTGCT	
rat-circ-Tlk2-LF	GTGTGCTAGCCCCGAGTAAG	175
rat-circ-Tlk2-LR	GAAGCATTCACAAGCGGAGC	
rat-circ-Susd1-CF	AGACGTCTGTGCCACTTGC	129
rat-circ-Susd1-CR	ACCCCCTAGGGAAACTTACCA	
rat-circ-Susd1-LF	GACACCTGTGTGAGATGGCA	190
rat-circ-Susd1-LR	TGCTCACGGTGTAATTGGCT	
rat-circ-Arl1-CF	CCATGTTGGAGGTGGCTTTT	133
rat-circ-Arl1-CR	AACTTCTCCAACCTGCAATCT	
rat-circ-Arl1-LF	CGACGAGGCAATGGAATGGT	136
rat-circ-Arl1-LR	CCTTCCACCAGTCACGAGAA	
rat-circ-Tcaim-CF	CGCAGTTGATGGATGCTTGG	125
rat-circ-Tcaim-CR	TCGATACATAACAGGGCGGAC	
rat-circ-Tcaim-LF	TGTCCACCATCACTGGACAC	140
rat-circ-Tcaim-LR	CAGGCTGCAGCTCTTCGATA	
rat-circ-Tbc1d8-CF	TGATCGATGCTGTGACGGAC	197
rat-circ-Tbc1d8-CR	CCGGTGAGCATAAGCTGTCA	
rat-circ-Tbc1d8-LF	AACGTTGGAGGAAATCAACCG	125
rat-circ-Tbc1d8-LR	CGATCAGAGCCTTCACCTTCC	
rat-circ-Phkb-CF	GCAGGCTGATAAGGCTCAGTT	183
rat-circ-Phkb-CR	GGACTTCTGATCTCCACCACAA	
rat-circ-Phkb-LF	TGCTCTACAAGCAGTCAGGC	165
rat-circ-Phkb-LR	GATCCTCCCTGGCAAACACA	
rat-Mmp9-F	CACTGTAACTGGGGGCAACT	150
rat-Mmp9-R	CACTTCTTGTCAGCGTCGAA	
rat-CCL2-F	ttcacagttgctgcctgtag	139
rat-CCL2-R	tgctgctggtgattctcttg	
rat-GAPDH-F	GCAAGAGAGAGGCCCTCAG	74
rat-GAPDH-R	TGTGAGGGAGATGCTCAGTG	

### siRNA and plasmid transfection

We synthesized circ-Phkb siRNA (siRNA-1, sense, 5′-AGGCTGATAAGGCTCAGTTTA-3′, antisense, 5′-TAAACTGAGCCTTATCAGCCT-3′;siRNA-2, sense, 5′-GCTGATAAGGCTCAGTTTATG-3′, antisense, 5′-CATAAACTGAGCCTTATCAGC-3′) (Genepharm, Shanghai, China) and add transfected them into cells using Lipofectin transfection reagent (Invitrogen) following the manufacture’s instruction. A lentivirus-based vector containing circ-Phkb fragment was constructed and transfected into cells using Lipofectin transfection reagent to overexpress circ-Phkb in NR8383 cells.

### Cell proliferation assay

Cell proliferation was determined by Cell Counting Kit-8 (CCK8) assay or 5-Ethynyl-2-deoxyuridine (Edu) staining. NR8383 cells were plated in a 96-well plate at a concentration of 3 × 10^4^ cells/mL (100 µL/well). Following LPS incubation for 24, 48, and 72 h, 10 µl of CCK-8 was added to each well, and the mixture was incubated for 2 h at 37 ℃. We used an auto-microplate reader (ReadMax 1900, Shanpu, Shanghai, China) to measure optical density at 450 nm. Cells were cultured in 24-well plates for the EdU assay. 10 µM EdU reagent (Biyotime, Shanghai, China) was added to each well and incubated at 37℃ for 2 h. Then, the cells were fixed with 4% paraformaldehyde, 0.3% Triton X-100 and incubated with 100 µl Click reaction mixture for 30 min. Finally, cell nuclei were stained with Hoechst-33,342 and images were captured using a fluorescence microscope.

### Flow cytometry

We collected NR8383 cells with EDTA-free pancreatin (Gibco) and washed them with PBS. Cells (1 × 10^6^ cells) were then resuspended in binding buffer, and 1 × 10^5^ cells were subsequently stained with Annexin V-FITC and PI (Sigma–Aldrich) for 20 min. Afterward, cells were incubated in the dark for 1 h at room temperature. We used FACScan (Beckman Coulter, Fullerton, CA, USA) to detect the population of apoptotic cells and FlowJo software (Treestar, Ashland, OR, USA) to analyze them.

### Terminal deoxynucleotidyl transferase UTP nick end labeling (TUNEL) assay

The cells were fixed with 4% PFA at room temperature for 1 h, permeabilized with 0.1% Triton for 10 min, washed with PBS, and stained with a commercial TUNEL cell death detection kit (Roche, Switzerland) according to the manufacturer’s instructions. We used a 200× inverted fluorescent microscope (Olympus, Tokyo, Japan) to measure the number of cells. Three fields of view per region were used to tally the number of TUNEL-positive cells.

### Western blot

Proteins were extracted from ALI lung rats or NR8383 cells using radioimmunoprecipitation assay lysis buffer (Beyotime). A total of 30 µg of proteins from each sample were separated using 10% sodium dodecyl sulfate-polyacrylamide gel electrophoresis. After transferring the proteins to the polyvinylidene fluoride (PVDF) membranes (Millipore, Bedford, USA), the PVDF membranes were blocked with 5% BSA (Beyotime), and then incubated with primary detection antibodies followed by anti-rabbit or anti-mouse secondary antibodies. Table 2 lists the antibodies used in this study. GAPDH was used as an internal loading control.

**Table 2:** The information of antibodys used in this study

Antibody name	Company	Product No.	Concentration of use
GAPDH	Proteintech	60004-1-lg	1: 8000
Caspase3	CST	9662	1: 1000
P65	Abcam	ab7970	1: 1000
Bax	Proteintech	50599-2-lg	1: 8000
TLR4	Abcam	ab217274	1: 300
Cleaved-Caspase3	CST	9661	1: 1000
CCL2	bioss	bs-1101R	1: 500
p-P65	CST	3033	1: 1000
MyD88	CST	4283	1: 1000
Bcl2	CST	3498	1: 1000

### Statistical analysis

All the data presented were analyzed with GraphPad Prism software v8.0. Data were expressed as mean ± standard deviation calculated from at least three independent experiments. Differences between two groups and more than two groups were analyzed with a t-test and a one-way analysis of variance, respectively. Statistical significance was set at *P < 0.05.

## Results

### CircRNA and mRNA profiles are alert in LPS-induced ALI rats

**Fig. 1:**
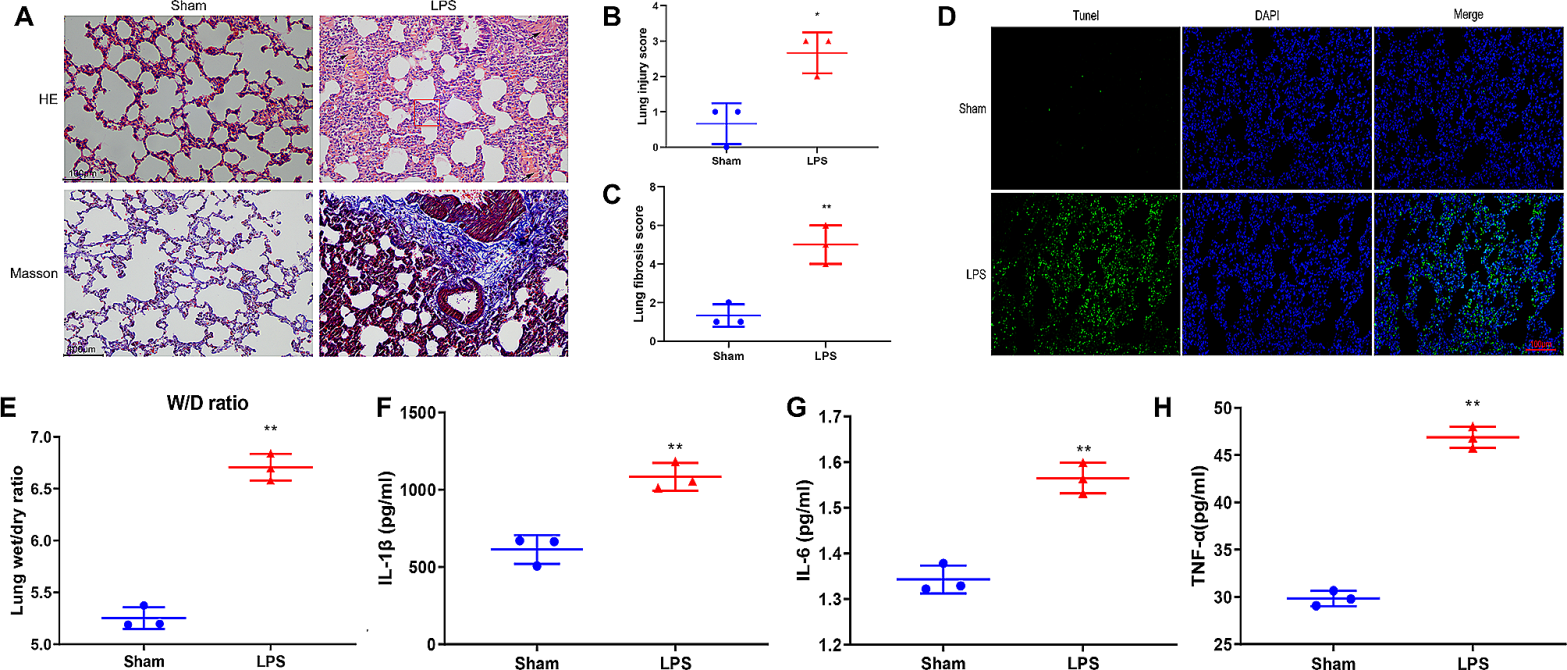
LPS-induced tissue damage, fibrosis, and inflammation in the lung of ALI rats. **(A)** H&E staining and Masson staining of Sham rats and ALI rats induced by LPS. The red boxes indicate the thickening of the alveolar septum, the black arrows represent bleeding, and the yellow arrows denote inflammatory cell infiltration. Lung injury score **(B)** and fibrosis score **(C)** in each group. **(D)** TUNEL assay showing apoptotic cells in lung tissue. **(E)** Lung wet/dry (W/D) ratio of each group. IL-1β **(F)**, IL-6 **(G)**, and TNF-α **(H)** inflammatory cytokines were measured by ELISA. ** *P* < 0.01 vs. Sham,* *P* < 0.05 vs. Sham

We developed the LPS-induced ALI rat model to investigate the circRNA and mRNA profiles. H&E and Masson’s staining revealed significant bleeding, alveolar septal thickening, inflammatory cell infiltration, and diffuse fibrosis in LPS-induced ALI rats (LPS) (Fig. 1A). Lung injury score and fibrosis score in the LPS group were significantly higher than those in the Sham rats (Sham) (Fig. 1B and C). Additionally, the TUNEL assay demonstrated an apoptotic signal in the lung tissue of rats, indicating a significantly increased number of apoptotic cells in ALI rats (Fig. 1D). Moreover, a higher lung W/D ratio was observed in the LPS group than in the Sham group (Fig. 1E). The contents of inflammatory cytokines IL-1β, IL-6, and TNF-α in alveolar lavage fluid were significantly higher than those in the Sham group (Fig. 1F and H).

**Fig. 2:**
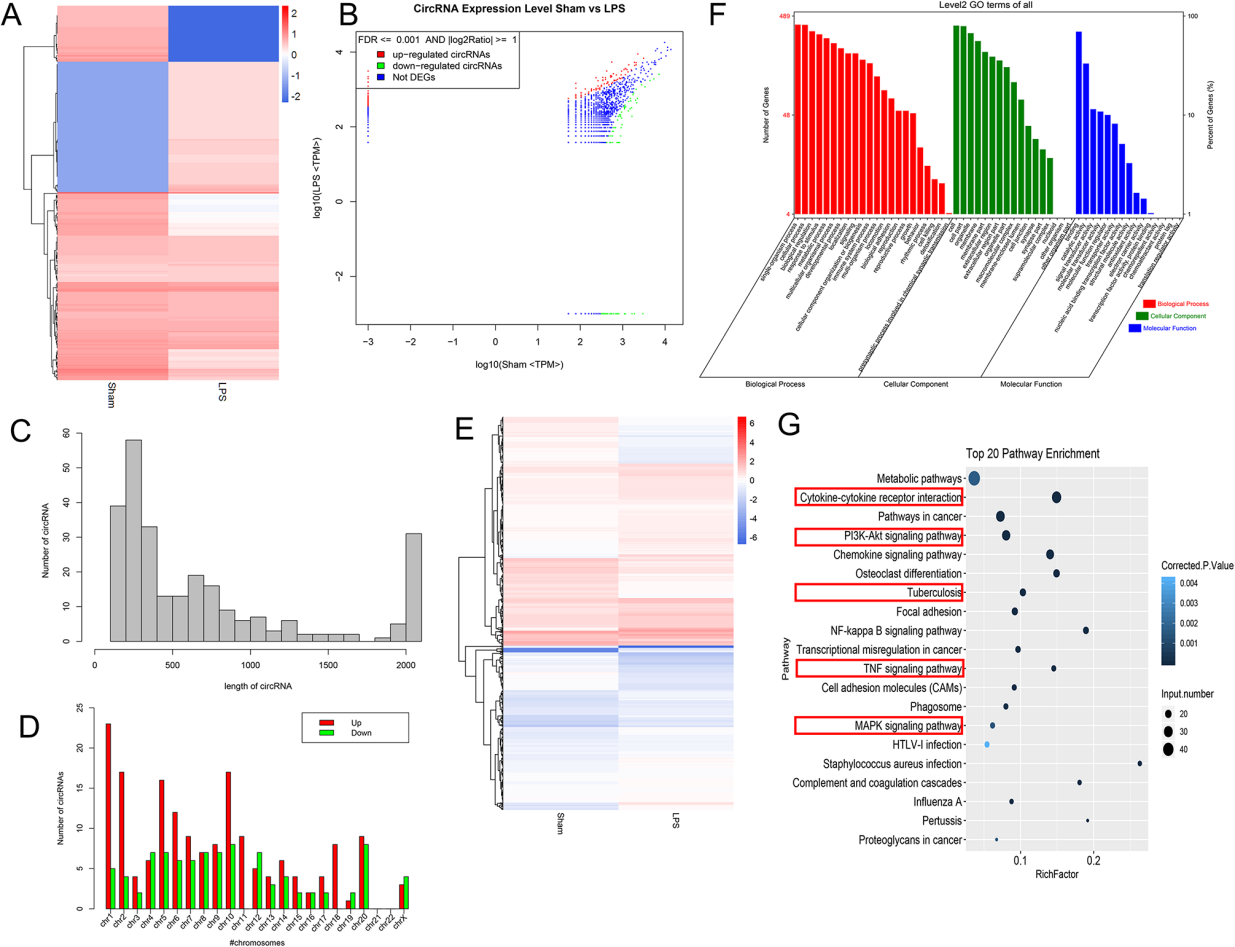
RNA-seq shows differential expression profiles of circRNA and mRNA in the lung tissue of ALI rats. **(A)** The heat map shows the differentially expressed circRNA expression profile of the LPS-induced ALI rats. **(B)** Scatter plot showing the differentially expressed circRNA. The length distribution **(C)** and the chromosome position of the differentially expressed circRNA **(D)**. **(E)** The heat map shows the differentially expressed mRNA expression profile of the LPS group. **(F)** GO analysis shows the most enriched GO terms for differentially expressed mRNA. **(G)** KEGG analysis shows the pathways enriched with the differentially expressed mRNAs

We collected lung tissues of LPS and Sham rats for RNA-seq sequencing and then analyzed the characteristics of differentially expressed circRNA and mRNA. The heat map showed that the circRNA expression profile of LPS was different from that of the Sham group (Fig. 2A). Scatter plots demonstrated significant changes in a series of circRNAs in the lungs of rats after LPS induction. Among them, 267 were differential circRNAs, 174 of which were upregulated in the LPS group and 93 were downregulated in the LPS-induced ALI rats (Fig. 2B). Analysis of the length of these differentially expressed circRNAs revealed that the main length of these circRNAs was concentrated within 500 nt (Fig. 2C). The genomic location analysis showed that these circRNAs were distributed on all chromosomes except for CHR21 and CHR22 (Fig. 2D).

We first analyzed the differences in transcriptome expression profiles in lung tissues of LPS and Sham rats to explore the potential function of differentially expressed circRNAs. The heat map demonstrated different transcriptome expression profiles of lung tissues of LPS and Sham rats. LPS rats demonstrated 689 differential mRNAs, 451 of which were upregulated in the LPS group and 238 were downregulated in the LPS group (Fig. 2E). GO analysis revealed that differentially expressed genes were enriched in terms of response to stimulus, immune system, metabolic, etc. (Fig. 2F). KEGG enrichment analysis revealed that differentially expressed genes were mainly concentrated in the signaling pathways of cytokine-cytokine receptor interaction, PI3K-Akt, tuberculosis, NF-kB, TNF, MAPK, etc. (Fig. 2G).

**Fig. 3:**
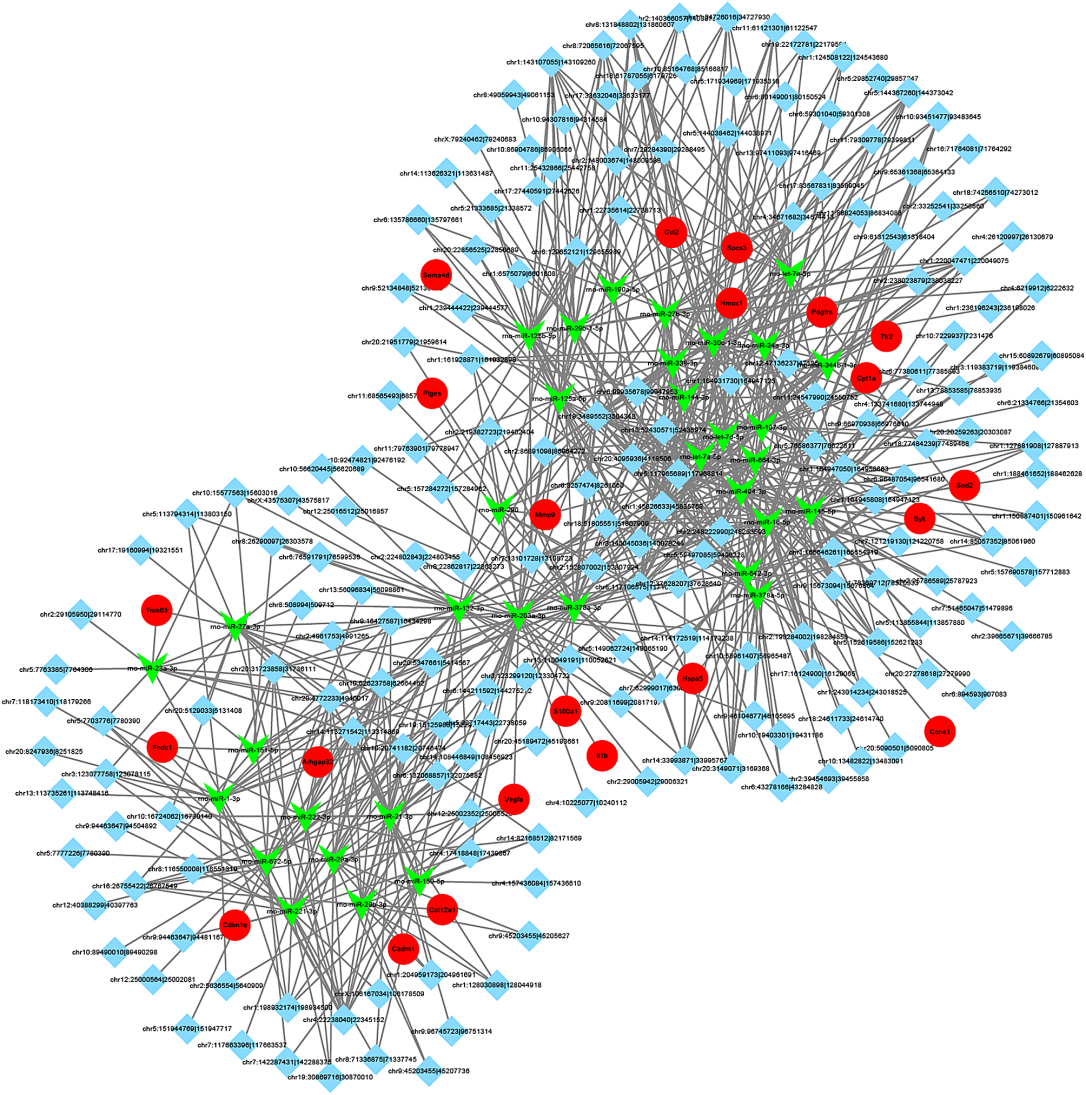
The ceRNA network for differentially expressed circRNAs and mRNAs

We further analyzed the correlation between differentially expressed circRNAs and differentially expressed mRNAs. We predicted the miRNAs that target both differentially expressed circRNAs and differentially expressed mRNAs, and then drew the circRNA-miRNA-mRNA interaction network (Fig. 3). We selected the top 8 circRNAs whose target genes were involved in inflammatory or oxidative damage signaling pathways and had the highest differential expression folds as candidates for further validation.

### Circ-phkb induces cell apoptosis and inflammation in lung macrophage

We stimulated rat lung macrophage cell line NR8383 with LPS (100 ng/ml) to develop an in vitro ALI model to study the function of differentially expressed circRNA on lung injury, and cell apoptosis, proliferation, and inflammatory factor levels were determined to assess the effects of LPS on cell injury and inflammation.

**Fig. 4:**
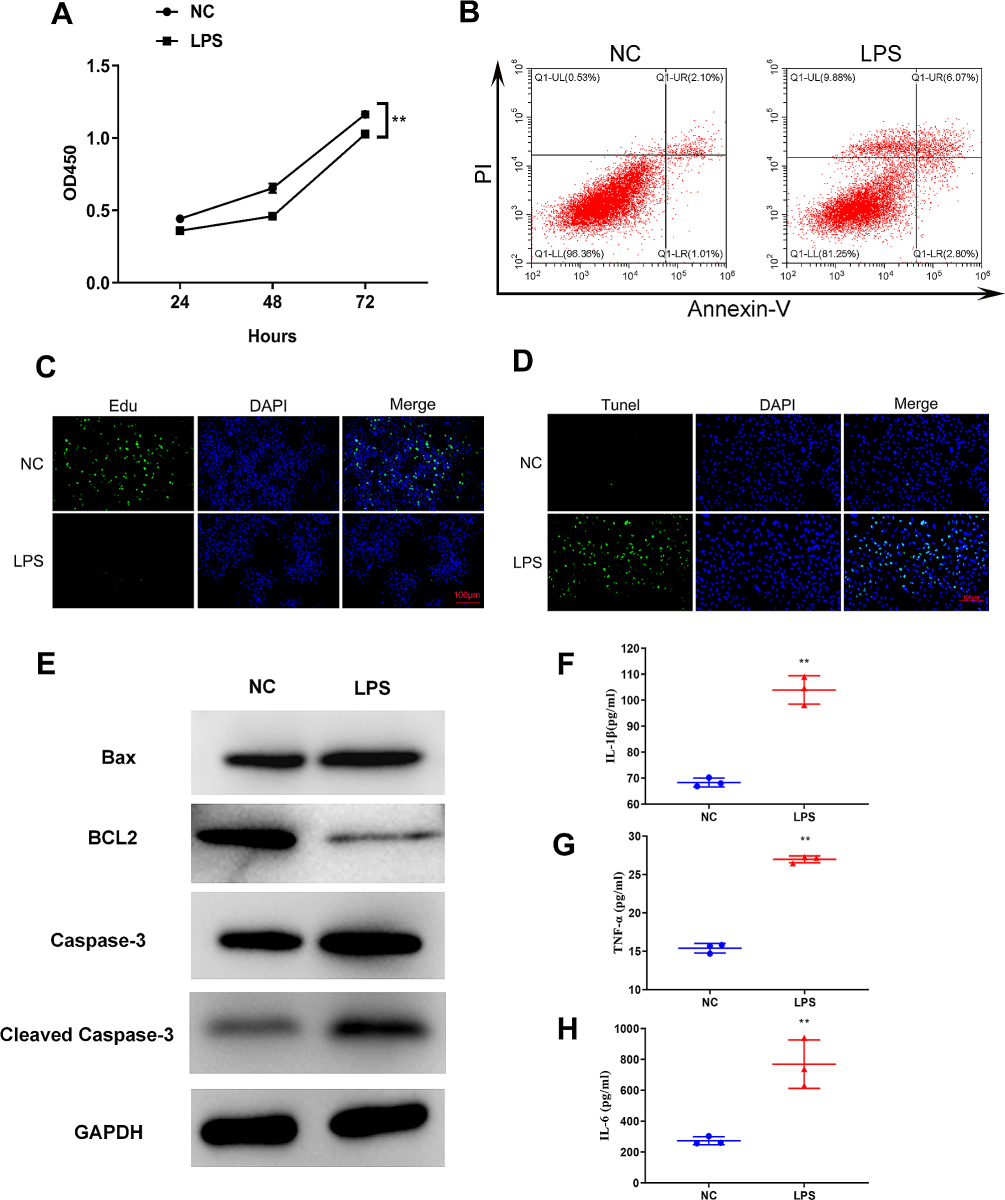
The effect of LPS on proliferation, apoptosis, and inflammation in NR8383 cells. **(A)** CCK8 assay showing the activity of LPS-induced NR8383. **(B)** Flow cytometry analysis of LPS-induced apoptosis of NR8383 after 48 h. **(C)** Edu staining showing the proliferation level of NR8383 cells after 48 h of LPS induction. **(D)** TUNEL assay showing the level of apoptosis in LPS-induced NR8383 cells. **(E)** WB showing the expression level of apoptosis-related protein. The levels of IL-1β **(F)**, TNF-α **(G)**, and IL-6 **(H)** in cell supernatant were detected by ELISA assay. **p < 0.01 vs. NC

Cell activity was first evaluated using CCK8 assay at 24 h, 48 h, and 72 h after LPS induction. A significant decrease in cell viability was observed after 48 h of LPS treatment (Fig. 4A) while the amount of apoptotic cells, which were detected by flow cytometry, were increased after 48 h of LPS induction (Fig. 4B). Moreover, the Edu assay revealed a suppressed cell proliferation in LPS-induced cells (Fig. 4C) whereas the TUNEL assay demonstrated increased apoptosis in LPS-induced cells for 48 h (Fig. 4D). Supported by flow cytometry and TUNEL assay results, Western blot (WB) revealed that apoptosis-associated proteins Bax and Caspase 3 were significantly upregulated while anti-apoptosis-associated BCL2 was significantly downregulated (Fig. 4E) after 48 h of LPS treatment. We determined the expression of inflammatory cytokines IL-1β, IL-6, and TNF-α in the supernatant of NR8383 cells to evaluate the effect of inflammation caused by LPS. ELISA assay revealed that the release of IL-1β, IL-6, and TNF-α was upregulated after 48 h of LPS induction (Fig. 4F and H).

**Fig. 5:**
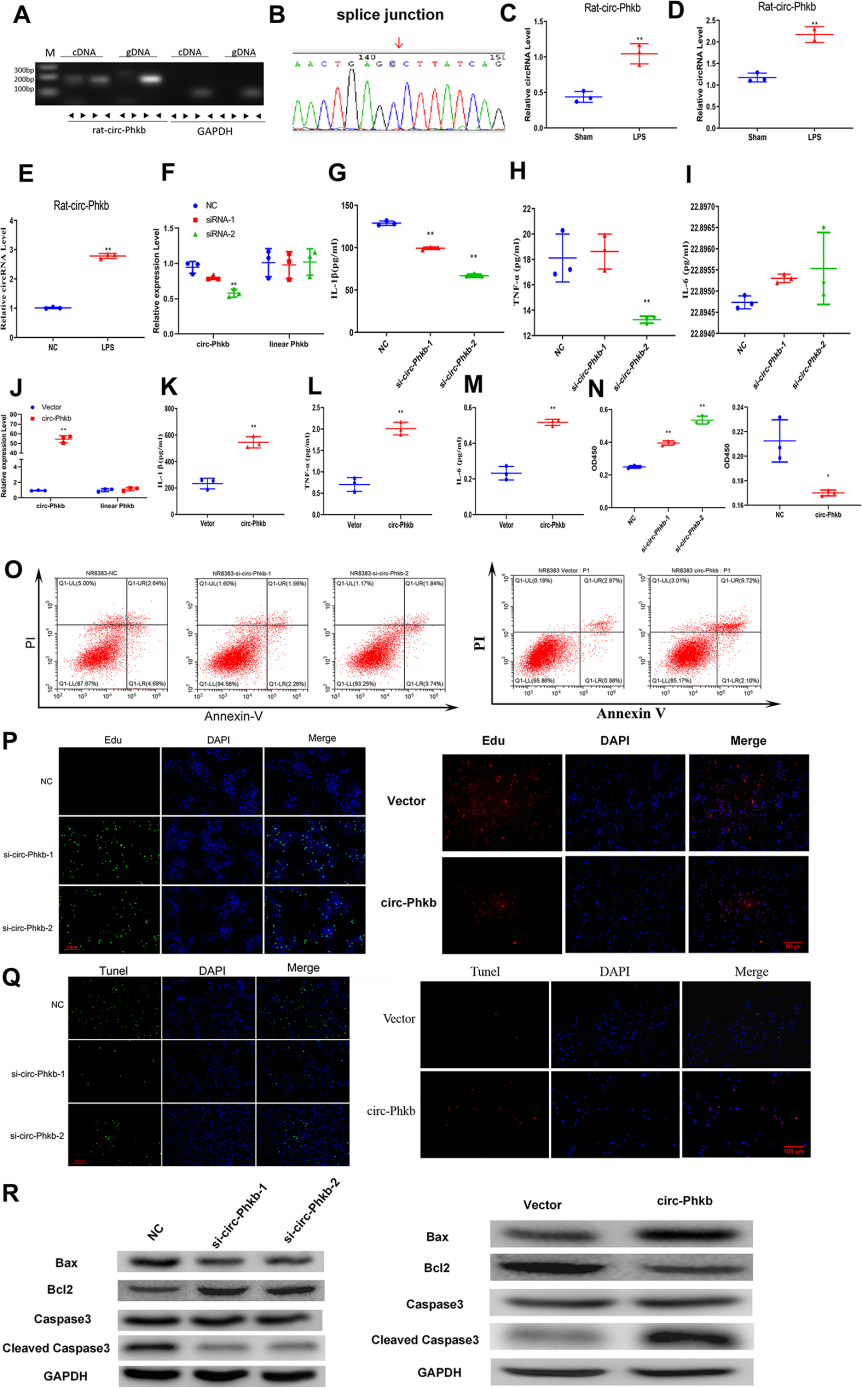
Circ-Phkb affects the expression of inflammatory factors and cell viability in rat alveolar macrophages. **(A)** PCR identification of the circular form of circ-Phkb. **(B)** Sanger sequencing verifying the splice junction of circ-Phkb. **(C)** The upregulated circ-Phkb expression in the lung tissues and lung macrophages of LPS-induced rats **(D)** and LPS-induced NR8383 cells **(E)** verified by qRT-PCR. **(F)** qRT-PCR showed that siRNAs significantly reduced the expression of circ-Phkb but not linear Phkb in NR8383 cells. ELISA assay showing the effects of circ-Phkb downregulation on the release of IL-1β **(G)**, TNF-α **(H)**, and IL-6 **(I)** in the supernatant of LPS-induced NR8383 cells. **(J)** qRT-PCR confirmed that circ-Phkb but not linear Phkb expression was significantly upregulated in NR8383 cells after transfection with circ-Phkb overexpression plasmid. ELISA assay showing the effects of circ-Phkb overexpression on the release of IL-1β **(K)**, TNF-α **(L)**, and IL-6 **(M)** in the supernatant of NR8383 cells. **(N)** CCK8 assay was used to detect the cell viability changes before and after inhibiting or promoting the circ-Phkb expression in NR8383 cells with LPS treatment. **(O)** Flow cytometry analysis was used to detect the cell apoptosis before and after inhibiting or promoting the circ-Phkb expression in NR8383 cells with LPS treatment. **(P)** Edu staining showing the proliferation level before and after inhibiting or promoting the circ-Phkb expression in NR8383 cells with LPS treatment. **(Q)** TUNEL assay showing the level of apoptosis alternation before and after inhibiting or promoting the circ-Phkb expression in NR8383 cells with LPS treatment. **(R)** WB assay showing the expression level of apoptosis-related protein changes before and after inhibiting or promoting the circ-Phkb expression in NR8383 cells with LPS treatment. **p < 0.01 vs. Sham/NC/Vector, *p < 0.05 vs. NC

We further confirmed 8 circRNAs in the LPS-induced in vitro model. Among them, circ-Phkb had a circular form and a cleavage site (Figures S1, S2, 5 A, and 5B), and were upregulated in lung tissues of LPS-induced rats (Figures S3 and 5 C) and alveolar macrophages (Fig. 5D) and alveolar macrophage NR8383 cells (Figures S4 and 5E). Additionally, the expression trend of circ-Phkb was consistent with the sequencing results, and the differential expression folds were higher between groups compared to other differential circRNAs, so it was selected for subsequent studies.

We further transfected circ-Phkb siRNA (siRNA-1 and siRNA-2) into NR8383 cells and subjected them to 24-h LPS treatment to clarify the function of circRNA. The cell supernatant was collected for ELISA assay. As shown in Fig. 5, circ-Phkb siRNA, both siRNA-1 and siRNA-2 significantly downregulated the expression of circ-Phkb and IL-1β, but they did not affect linear Phkb mRNA expression (Fig. 5F and G). SiRNA-2 can significantly down-regulate the expression of TNF-α but has no significant effect on IL-6 expression (Fig. 5H and I). On the contrary, the expression of circ-Phkb was significantly increased after the circ-Phkb plasmid was transfected into NR8383 cells for 24 h while linear Phkb mRNA expression remained unchanged (Fig. 5J). ELISA assay revealed that circ-Phkb overexpression significantly up-regulates the expression of IL-1β, TNF-α, and IL-6 (Fig. 5K and M). Additionally, circ-Phkb inhibition in NR8383 cells with LPS treatment increased the cell viability and anti-apoptosis-related protein expression (BCL2), decreased the apoptosis rate and pro-apoptosis-related protein expression (Bax, cleaved caspase 3), but circ-Phkb overexpression had the opposite effect (Fig. 5N and R).

### Circ-phkb promotes CCL2 expression via TLR4/MyD88/NF-kB pathway in rat alveolar macrophages

**Fig. 6:**
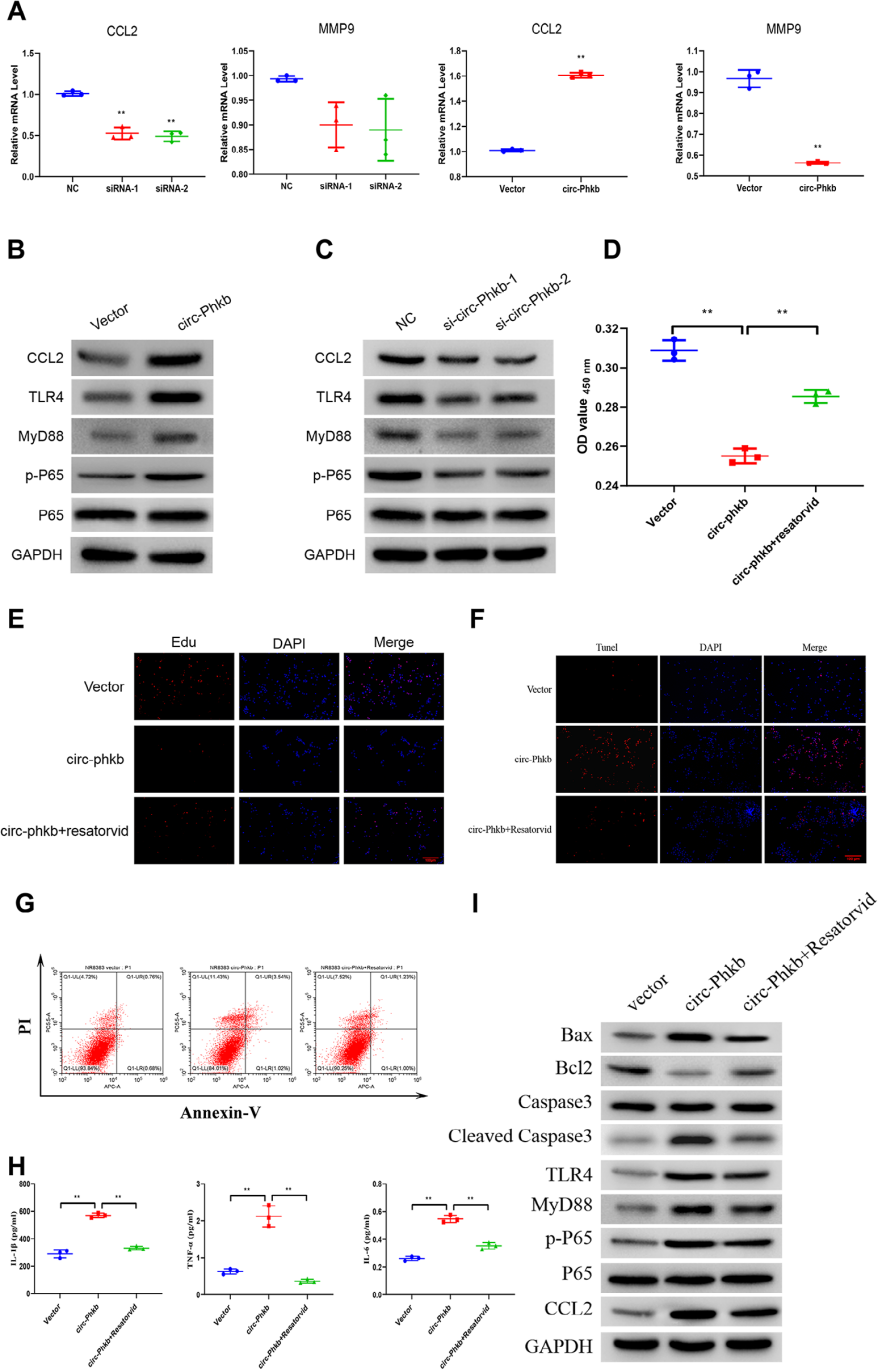
Circ-Phkb affects the expression and function of CCL2 by regulating TLR4/MyD88/NF-kB signaling. **(A)** qRT-PCR showing the effect of circ-Phkb downregulation and overexpression on CCL2 and MMP9 expressions. WB showing the effects of circ-Phkb overexpression **(B)** and downregulation **(C)** on the TLR4/MyD88/NF-kB signaling pathway. CCK8 assay **(D)**, Edu staining **(E)**, TUNEL assay **(F)**, flow cytometry **(G)**, and ELISA assay **(H)** showing the effect of TLR4 signaling pathway inhibitor (Resatorvid) on proliferation, apoptosis, and levels of IL-1β, TNF-α, and IL-6 in circ-Phkb that overexpresses NR8383 cells after 24 h of Resatorvid treatment. **(I)** WB shows the effect of Resatorvid on the TLR4/MyD88/NF-kB signaling pathway, apoptosis-associated proteins, and CCL2 expression in NR8383 cells overexpressing circ-Phkb. **p < 0.01 vs. NC/Vector/circ-Phkb

Bioinformatics indicates that CCL2 and MMP9 are the downstream target genes of circ-Phkb. We used qPCR to detect the effects of circ-Phkb downregulation and overexpression on the mRNA and protein levels of CCL2 and MMP9 in NR8383 cells to verify whether circ-Phkb regulates CCL2 and MMP9. Transfection of circ-Phkb siRNA-1 and siRNA-2 significantly inhibited CCL2 mRNA expression but did not affect MMP9 mRNA level (Fig. 6). On the contrary, the transfection of circ-Phkb overexpressing plasmid significantly upregulated the mRNA level of CCL2 and downregulated the mRNA level of MMP9 (Fig. 6A).

Abnormal TLR4/MyD88/NF-kB pathway activation is one of the important disease mechanisms of ALI. We transfected circ-Phkb siRNA and overexpression plasmid into cells and detected the expression of key proteins in the pathway to detect the function of circ-Phkb on TLR4/MyD88/NF-kB pathway and circ-Phkb. WB results revealed that circ-Phkb expression increased TLR4 and MyD88 expression and P65 phosphorylation in the NF-kB pathway (Fig. 6B). In contrast, circ-Phkb siRNA-1 and siRNA-2 significantly inhibited the TLR4 and MyD88 expressions and the P65 phosphorylations (Fig. 6C) after LPS induction. We further investigated whether the TLR4 pathway inhibitor Resatorvid has an antagonistic effect on the cellular biological functions of circ-Phkb to further confirm that the effect of circ-Phkb on cells is related to this pathway.

CCK8 assay revealed that Resatorvid partially reverses the decrease in cell viability (Fig. 6D) and cell proliferation caused by circ-Phkb expression, which can be proved from the results of Edu staining (Fig. 6E). Simultaneously, both TUNEL assay and cytometry revealed that Resatorvid inhibited the apoptosis level caused by circ-Phkb overexpression in NR8383 cells (Fig. 6F and G). Additionally, the ELISA results revealed that Resatorvid inhibited the increase of IL-1β, TNF-α, and IL-6 that were induced by circ-Phkb overexpression (Fig. 6H). Finally, the WB results demonstrated that the circ-Phkb overexpression caused the upregulation of pro-apoptotic-related proteins Bax, cleaved-Caspase3, and CCL2, while the downregulation of the apoptosis-inhibiting protein BCL2, and the activation of the key protein of TLR4/MyD88/NF-kB pathway, including TLR4, MyD88, p-P65, and p65 (Fig. 6I).

## Discussion

The existing research on the expression and function of circRNA in ALI remains limited. Previous studies have revealed that the expression profile of circRNA in the lungs of the ALI mouse model induced by LPS has significantly changed. Additionally, these differentially expressed circRNAs it is predicted to regulate metabolic pathways such as cellular macromolecule metabolic process and cellular metabolic process [12]. Another study revealed that circRNA is ubiquitous and differentially expressed in the lungs of rats with ALI caused by smoke inhalation [11]. Similar to the results of these previous studies, our study revealed differentially expressed circRNAs based on the LPS-induced rat model. Our results and other studies indicate that the expression changes of circRNA in ALI may be a common phenomenon and may be involved in regulating the pathological process of ALI.

Lung macrophages, mainly alveolar macrophages, are one of the key factors in ALI occurrence and development [17, 18]. Studies have revealed that lung alveolar macrophage depletion reduces pulmonary edema, lung inflammation, and lung injury in animal models of lung injury [8, 19]. Additionally, previous studies have revealed the expression profile of circRNA in lung macrophages of the ALI mouse model through high-throughput sequencing and predicted its possible biological functions [10]. However, the specific functions of these differentially expressed circRNAs remain unclear. Compared with previous studies, our results provide the expression profile of lung circRNA in the LPS-induced rat ALI model, and we explored the function of circ-Phkb in alveolar macrophages based on these results.

Glycogen phosphorylase kinase β-subunit (PHKB) is the regulatory subunit of phosphorylase kinase, which functions to activate glycogen phosphorylase and glycogen decomposition. It inhibits the proliferation and metastasis of liver cancer cells and promotes apoptosis in tumors [20]. PHKB has different circular RNA isoforms, but their function is unknown. The existing knowledge reported that circ-Phkb-2 has been highly enriched in adipocytes [21]. Additionally, Phkb-derived circRNA upregulation is observed in serum exosomes of patients with idiopathic membranous nephropathy [22]. Cells that are genetically programmed to die in a controlled, ordered manner to preserve the integrity of the internal environment undergo apoptosis. Several genes are activated, expressed, and regulated during apoptosis [23]. Alveolar macrophages are very important for maintaining homeostasis in the airways as the core cells of lung immunomodulation and are involved in the development of a variety of pulmonary diseases [6]. Reduced numbers of alveolar macrophages by apoptosis reduce their ability to phagocytose inflammatory effector cells, which causes the programmed release of large amounts of inflammatory mediators from inflammatory cells, thereby exacerbating lung injury [18]. Our study revealed that Phkb gene-derived circ-Phkb inhibits survival while promoting apoptosis in alveolar macrophages. This function is similar to that of the parent gene PHKB, which is involved in inhibiting cell survival. On the one hand, our results revealed evidence that circ-Phkb regulates lung macrophage survival, on the other hand, it also demonstrated some clues to the function of the cyclic form of PHKB and its parent gene.

This study revealed that circ-Phkb promotes the expression of pro-inflammatory factor (IL-1β, TNF-α, and IL-6) release. Various extracellular signals, such as LPS, stimulate TLR4 to activate MyD88 [24, 25], and then promote the translocation of transcription factor NF-κB to the nucleus, which is the key way for expressing inflammatory factors, including IL-1β, TNF-α, and IL-6 [26]. Research evidence has revealed an abnormally activated TLR4 pathway in ALI and an inhibited TLR4 pathway in ALI animals that have remission after drug treatment [27, 28]. These findings indicate that TLR4 pathway activation plays an important role in ALI. Our study revealed that differentially expressed mRNA is enriched in cytokine-cytokine receptor interaction, PI3K-Akt signaling pathway, TNF, MARK, and other inflammation-related signaling pathways. Additionally, we demonstrated the inhibitory effect of circ-Phkb inhibition on the TLR4/MyD88/NF-kB pathway, while its overexpression promotes pathway activation and the expression of downstream pro-inflammatory factors. These findings indicated that circ-Phkb promotes alveolar macrophage and ALI inflammation by promoting TLR4/MyD88/NF-kB.

We also revealed that circ-Phkb promotes CCL2 expression through the TLR4 pathway. CCL2 is an important regulator of chemotactic monocyte/macrophage transport in CCL2 circulating monocytes and interstitial macrophages. The macrophages are infiltrated into the site of injury or inflammation when infection or inflammation occurs [29]. CCR2 and CCR5 are CCL2 receptors. Increased CCL2 expression enhances the interaction between CCL2 and its receptor and enhances epithelial regeneration after lung injury [30, 31]. Therefore, CCL2 is a great therapeutic target in the pro-inflammatory of ALI. Our study revealed that circ-Phkb can promote CCL2 expression through the TLR4/MyD88/NF-kB pathway, as we have observed that TLR4 inhibitors attenuated the overexpression of key proteins of TLR4/MyD88/NF-kB and CCL2 caused by circ-Phkb overexpression. The promotion of CCL2 by circ-Phkb indicates that circ-Phkb may further inhibit the adhesion function of mononuclear macrophages in the inflammatory microenvironment because CCL2 functions as chemotaxis to mononuclear macrophages, which requires further research in the future.

In summary, we revealed that several circRNAs and mRNAs are differentially expressed in ALI rats. Among them, the upregulated circ-Phkb inhibits the proliferation of alveolar macrophages, thereby promoting apoptosis and the release of pro-inflammatory factors. One of the regulatory pathways is by promoting the TLR4/MyD88/NF-kB pathway. Circ-Phkb contributes to ALI and might be a potential target of ALI treatment.

## Conclusions

Rat circ-Phkb inhibits the survival of alveolar macrophages and increases inflammation, which is involved in promoting LPS-induced ALI. One of its possible mechanisms is through the TLR4/MyD88/NF-kB signaling pathway activation and CCL2 upregulation.

### Electronic supplementary material

Below is the link to the electronic supplementary material.

**Supplementary Material 1:** Supplemental figure legends

**Supplementary Material 2: Fig. 1.** The agarose gel electrophoresis of seven additional circRNAs

**Supplementary Material 3: Fig. 2.** The Sanger sequencing of seven additional circRNAs

**Supplementary Material 4: Fig. 3.** The expression of seven additional circRNAs in the lung tissues verified by qRT-PCR

**Supplementary Material 5: Fig. 4.** The expression of seven additional circRNAs in LPS-induced NR8383 cells verified by qRT-PCR

## Data Availability

The dataset generated and analysed during the current study is available in the Gene Expression Omnibus (GEO) repository, GSE226036 (enter secure: etuhcsgixnwbvwp).
