# Supplementary material for: Circ-phkb promotes cell apoptosis and inflammation in LPS-induced alveolar macrophages via the TLR4/MyD88/NF-kB/CCL2 axis
Source: Respir Res. 2024 Jan 29;25:62. doi: 10.1186/s12931-024-02677-6 (PMC10826187; doi:10.1186/s12931-024-02677-6)
Supplement: Supplementary file 1 — Supplementary Material 1: Supplemental figure legends [file 12931_2024_2677_MOESM1_ESM.docx]

Supplemental Figure 1. The agarose gel electrophoresis of 7 additional circRNAs.

Supplemental Figure 2. The sanger sequencing of 7 additional circRNAs.

Supplemental Figure 3. The expression of 7 additional circRNAs in the lung tissues was verified by qRT-PCR.

Supplemental Figure 4. The expression of 7 additional circRNAs in LPS induced NR8383 cells was verified by qRT-PCR.
