# Supplementary figures and images for: Circ-phkb promotes cell apoptosis and inflammation in LPS-induced alveolar macrophages via the TLR4/MyD88/NF-kB/CCL2 axis
Source: Respir Res. 2024 Jan 29;25:62. doi: 10.1186/s12931-024-02677-6 (PMC10826187; doi:10.1186/s12931-024-02677-6)

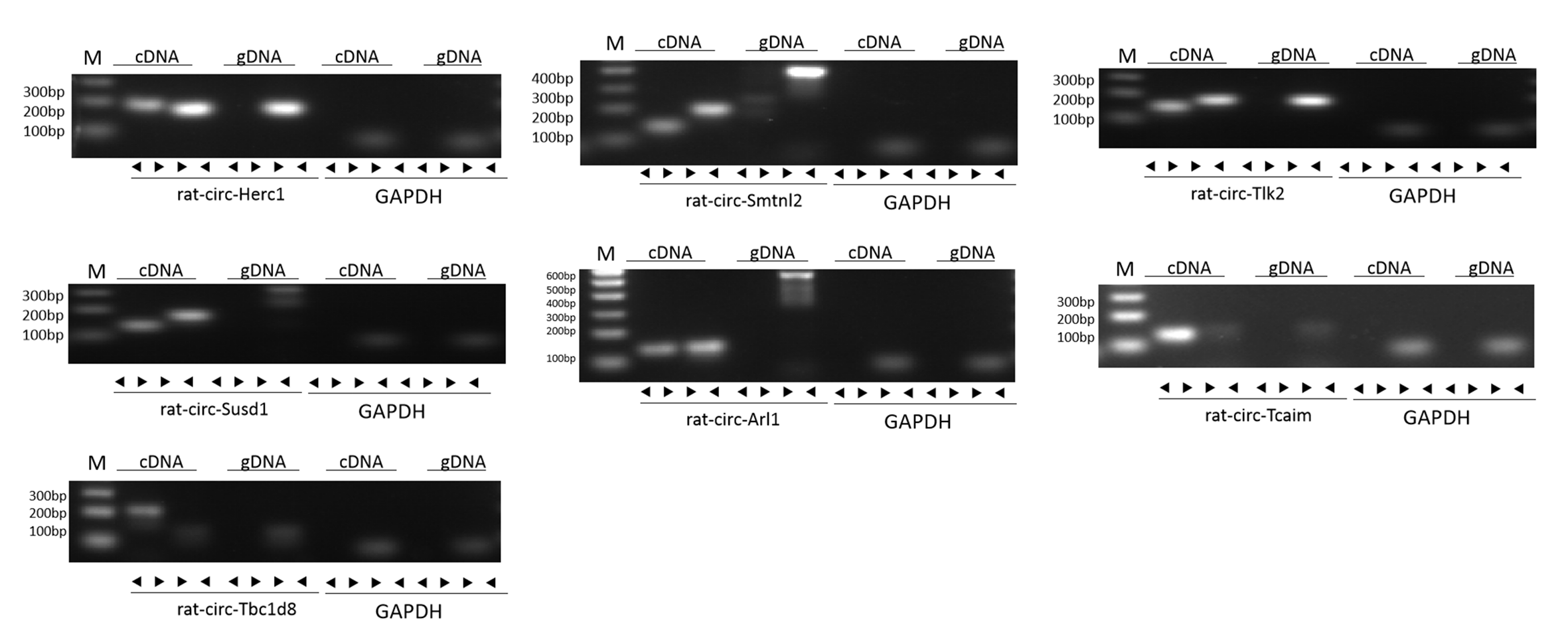

Supplement: Supplementary file 2 — Supplementary Material 2: Fig. 1. The agarose gel electrophoresis of seven additional circRNAs [file 12931_2024_2677_MOESM2_ESM.tif]

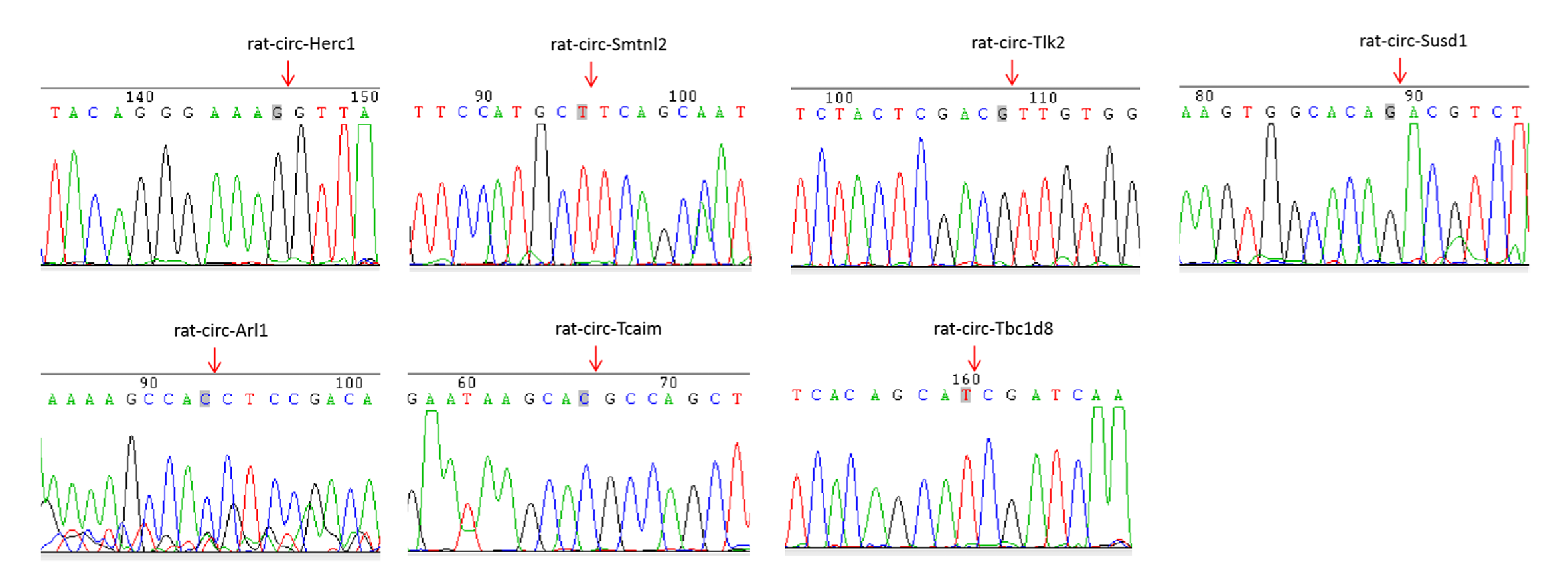

Supplement: Supplementary file 3 — Supplementary Material 3: Fig. 2. The Sanger sequencing of seven additional circRNAs [file 12931_2024_2677_MOESM3_ESM.tif]

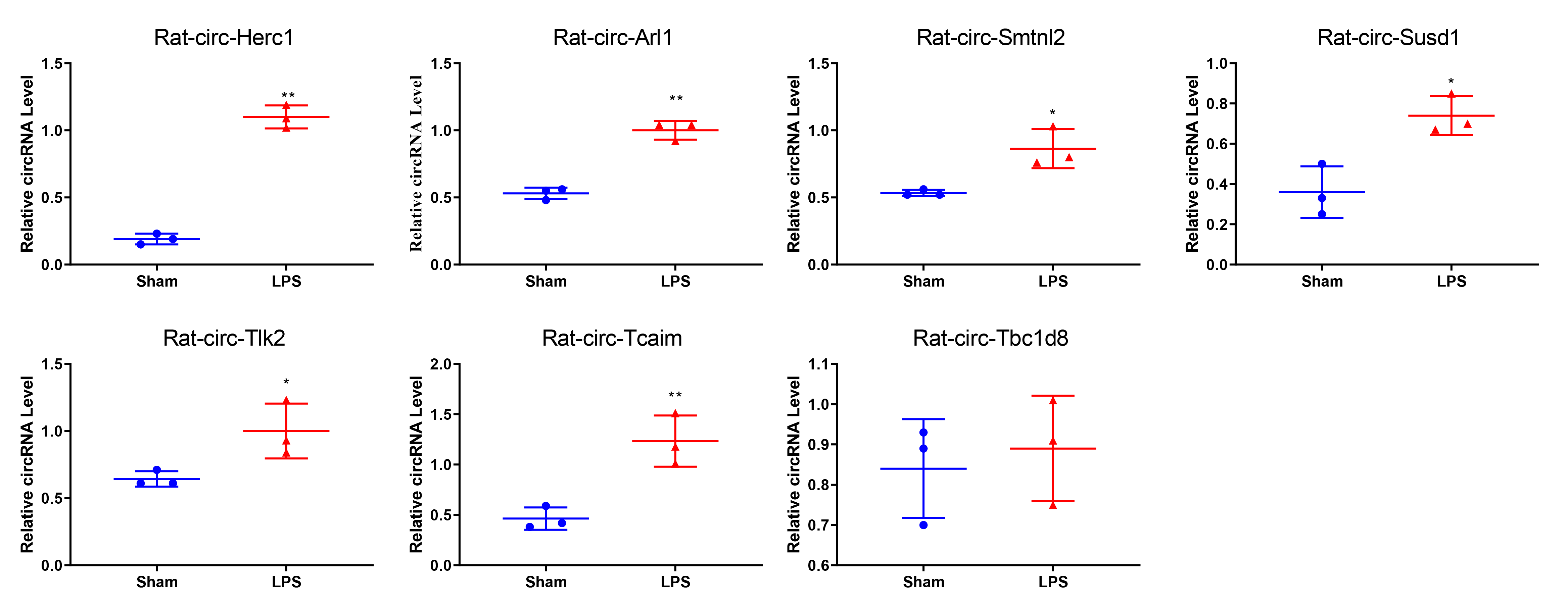

Supplement: Supplementary file 4 — Supplementary Material 4: Fig. 3. The expression of seven additional circRNAs in the lung tissues verified by qRT-PCR [file 12931_2024_2677_MOESM4_ESM.tif]

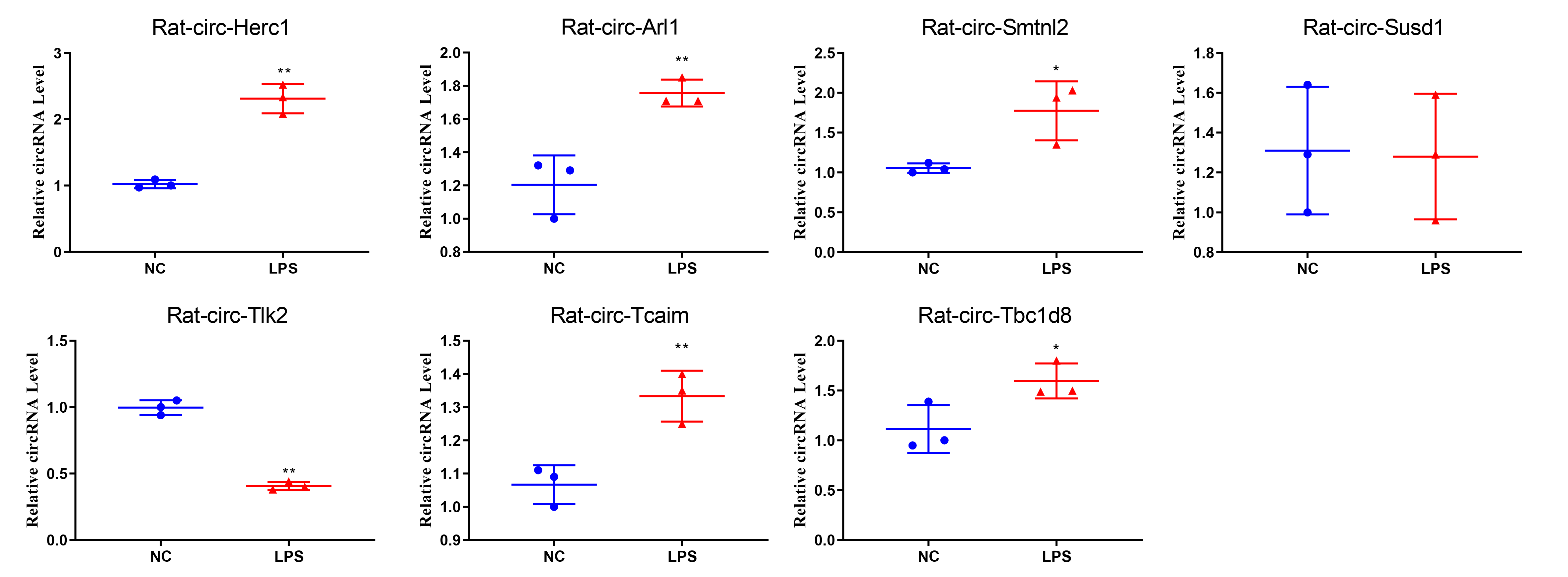

Supplement: Supplementary file 5 — Supplementary Material 5: Fig. 4. The expression of seven additional circRNAs in LPS-induced NR8383 cells verified by qRT-PCR [file 12931_2024_2677_MOESM5_ESM.tif]
